# (*S*)-1,2,4-Trimethyl­piperazine-1,4-diium tetra­chloridozincate(II)

**DOI:** 10.1107/S1600536810028631

**Published:** 2010-07-24

**Authors:** Zong-ling Ru

**Affiliations:** a Department of Chemical and Environmental Engineering, Anyang Institute of Technology, Anyang 455000, People’s Republic of China

## Abstract

In the title compound, (C_7_H_18_N_2_)[ZnCl_4_], the Zn atom adopts a slightly distorted tetra­hedral geometry. The diprotonated piperazine ring adopts a chair conformation. In the crystal structure, the cations and anions are linked by inter­molecular N—H⋯Cl hydrogen bonds into a chain along [001].

## Related literature

For the ferroelectric behavior of chiral coordination compounds, see: Fu *et al.* (2007 ▶). For non-linear optical second harmonic generation of chiral coordination compounds, see: Qu *et al.* (2003 ▶). For transition-metal complexes of (*S*)-2-methyl­piperazine, see: Ye *et al.* (2009 ▶). For puckering parameters, see: Cremer & Pople (1975 ▶). For hydrogen-bond motifs, see: Bernstein *et al.* (1995 ▶).
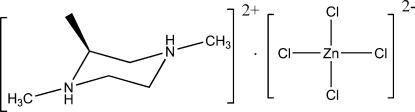


## Experimental

### 

#### Crystal data

(C_7_H_18_N_2_)[ZnCl_4_]
*M* *_r_* = 337.40
Orthorhombic, 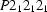

*a* = 8.5197 (17) Å
*b* = 9.7036 (19) Å
*c* = 17.013 (3) Å
*V* = 1406.5 (5) Å^3^
*Z* = 4
Mo *K*α radiation
μ = 2.48 mm^−1^
*T* = 293 K
0.30 × 0.28 × 0.26 mm

#### Data collection

Rigaku SCXmini diffractometer
Absorption correction: multi-scan (*CrystalClear*; Rigaku/MSC, 2005 ▶) *T*_min_ = 0.80, *T*_max_ = 0.90
14785 measured reflections
3217 independent reflections
2802 reflections with *I* > 2σ(*I*)
*R*_int_ = 0.038

#### Refinement

*R*[*F* ^2^ > 2σ(*F* ^2^)] = 0.032
*wR*(*F* ^2^) = 0.067
*S* = 1.08
3217 reflections
138 parameters
H atoms treated by a mixture of independent and constrained refinement
Δρ_max_ = 0.36 e Å^−3^
Δρ_min_ = −0.36 e Å^−3^
Absolute structure: Flack (1983 ▶), 1355 Friedel pairs
Flack parameter: 0.046 (14)

### 

Data collection: *CrystalClear* (Rigaku/MSC, 2005 ▶); cell refinement: *CrystalClear*; data reduction: *CrystalClear*; program(s) used to solve structure: *SHELXS97* (Sheldrick, 2008 ▶); program(s) used to refine structure: *SHELXL97* (Sheldrick, 2008 ▶); molecular graphics: *SHELXTL* (Sheldrick, 2008 ▶); software used to prepare material for publication: *SHELXL97*.

## Supplementary Material

Crystal structure: contains datablocks I, global. DOI: 10.1107/S1600536810028631/bx2289sup1.cif

Structure factors: contains datablocks I. DOI: 10.1107/S1600536810028631/bx2289Isup2.hkl

Additional supplementary materials:  crystallographic information; 3D view; checkCIF report

## Figures and Tables

**Table 1:** Hydrogen-bond geometry (Å, °)

*D*—H⋯*A*	*D*—H	H⋯*A*	*D*⋯*A*	*D*—H⋯*A*
N1—H1*A*⋯Cl1^i^	0.93 (3)	2.16 (3)	3.092 (2)	177 (3)
N2—H2*C*⋯Cl2^ii^	0.91 (3)	2.24 (3)	3.140 (3)	171 (3)

Symmetry codes: (i) 
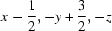
; (ii) 
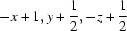
.

**Fig. 1.**
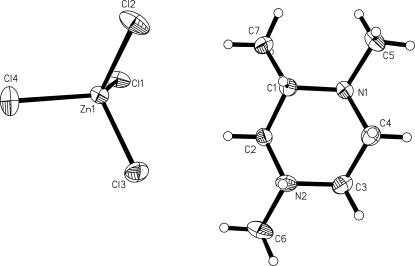
The asymmetric unit of the title compound with atom labels. Displacement ellipsoids were drawn at the 30% probability level

**Fig. 2.**
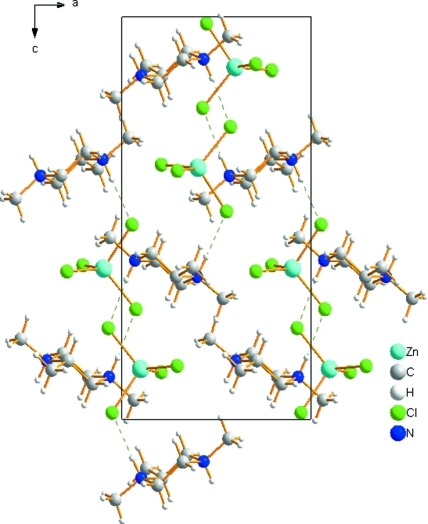
The packing viewed along the b-axis. Hydrogen bonds are drawn as dashed lines

## supplementary crystallographic information

### Comment

The existence of a chiral centre in an organic ligand is very important for the construction noncentrosymmetric or chiral coordination polymers that exhibit desirable physical properties such as ferroelectricity (Fu *et al.*, 2007) and nonlinear optical second harmonic generation (Qu *et al.*, 2003). Chiral (*S*)-2-methylpiperazine has a chiral centre which have shown tremendous scope in the synthesis of transition-metal complexes (Ye *et al.*, 2009). The construction of new members of this family of ligands is an important direction in the development of modern coordination chemistry. We report here the crystal structure of the title compound

The asymmetric unit of the title compound, (C_7_H_18_N_2_)[ZnCl_4_] (Fig.1), consists of one 1,2,4-trimethylpiperazinium cation and one  ZnCl_4_^2- ^anion. The Zn atom adopts a slightly distorted tetrahedral geometry. The diprotonated piperazine ring adopts a chair conformation with Cremer & Pople (1975) puckering parameters : Q_T_ =0.5673 (3)Å, θ = 1.8 (3)° , φ= 67 (10)°. In the crystal structure, cations and anions are linked by intermolecular N—H···Cl hydrogen bonds into a one-dimensional chain viewed along the c-axis with set graph-motif C_2_^2 ^(9) (Bernstein, *et al.*, 1995) (Fig.2).

### Experimental

A mixture of (*S*)-1,2,4-trimethylpiperazine quinine (1 mmol, 0.128 g), ZnCl_2_(1 mmol, 0.136 g) and 10% aqueous HCl (6 ml) were mixed and dissolved in 20 ml water by heating to 363 K (15 min) forming a clear solution. The reaction mixture was cooled slowly to room temperature, crystals of the title compound were formed after 8 days.

### Refinement

All H atoms were placed in calculated positions, with C—H = 0.93–0.98Å and N—H = 0.90 Å, and refined using a riding model, with *U*_iso_(H)=1.2*U*_eq_(C,*N*) or 1.5 *U*_eq_(C) for methyl H atoms.

### Crystal data

**Table:**

(C_7_H_18_N_2_)[ZnCl_4_]	*F*(000) = 688
*M**_r_* = 337.40	*D*_x_ = 1.593 Mg m^−^^3^
Orthorhombic, *P*2_1_2_1_2_1_	Mo *K*α radiation, λ = 0.71073 Å
Hall symbol: P 2ac 2ab	Cell parameters from 2802 reflections
*a* = 8.5197 (17) Å	θ = 3.2–27.5°
*b* = 9.7036 (19) Å	µ = 2.48 mm^−^^1^
*c* = 17.013 (3) Å	*T* = 293 K
*V* = 1406.5 (5) Å^3^	Block, colourless
*Z* = 4	0.30 × 0.28 × 0.26 mm

### Data collection

**Table:**

Rigaku SCXmini diffractometer	3217 independent reflections
Radiation source: fine-focus sealed tube	2802 reflections with *I* > 2σ(*I*)
graphite	*R*_int_ = 0.038
Detector resolution: 13.6612 pixels mm^-1^	θ_max_ = 27.5°, θ_min_ = 3.2°
ω scans	*h* = −11→10
Absorption correction: multi-scan (*CrystalClear*; Rigaku/MSC, 2005)	*k* = −12→12
*T*_min_ = 0.80, *T*_max_ = 0.90	*l* = −22→22
14785 measured reflections	

### Refinement

**Table:**

Refinement on *F*^2^	Secondary atom site location: difference Fourier map
Least-squares matrix: full	Hydrogen site location: inferred from neighbouring sites
*R*[*F*^2^ > 2σ(*F*^2^)] = 0.032	H atoms treated by a mixture of independent and constrained refinement
*wR*(*F*^2^) = 0.067	*w* = 1/[σ^2^(*F*_o_^2^) + (0.0257*P*)^2^ + 0.2767*P*] where *P* = (*F*_o_^2^ + 2*F*_c_^2^)/3
*S* = 1.08	(Δ/σ)_max_ = 0.001
3217 reflections	Δρ_max_ = 0.36 e Å^−^^3^
138 parameters	Δρ_min_ = −0.36 e Å^−^^3^
0 restraints	Absolute structure: Flack (1983), 1355 Friedel pairs
Primary atom site location: structure-invariant direct methods	Flack parameter: 0.046 (14)

### Special details

**Table:**

Geometry. All e.s.d.'s (except the e.s.d. in the dihedral angle between two l.s. planes) are estimated using the full covariance matrix. The cell e.s.d.'s are taken into account individually in the estimation of e.s.d.'s in distances, angles and torsion angles; correlations between e.s.d.'s in cell parameters are only used when they are defined by crystal symmetry. An approximate (isotropic) treatment of cell e.s.d.'s is used for estimating e.s.d.'s involving l.s. planes.
Refinement. Refinement of *F*^2^ against ALL reflections. The weighted *R*-factor *wR* and goodness of fit *S* are based on *F*^2^, conventional *R*-factors *R* are based on *F*, with *F* set to zero for negative *F*^2^. The threshold expression of *F*^2^ > σ(*F*^2^) is used only for calculating *R*-factors(gt) *etc*. and is not relevant to the choice of reflections for refinement. *R*-factors based on *F*^2^ are statistically about twice as large as those based on *F*, and *R*- factors based on ALL data will be even larger.

### Fractional atomic coordinates and isotropic or equivalent isotropic displacement parameters (Å^2^)

**Table:**

	*x*	*y*	*z*	*U*_iso_*/*U*_eq_	
Zn1	0.59768 (3)	0.49854 (3)	0.125016 (17)	0.03377 (9)	
C1	0.2131 (4)	0.8709 (3)	0.14589 (17)	0.0359 (7)	
H1	0.2576	0.8595	0.1986	0.043*	
C2	0.3442 (3)	0.8994 (3)	0.08782 (17)	0.0368 (7)	
H2A	0.4167	0.8223	0.0882	0.044*	
H2B	0.3000	0.9060	0.0354	0.044*	
C3	0.3220 (4)	1.1457 (3)	0.10626 (19)	0.0481 (9)	
H3A	0.2799	1.1599	0.0539	0.058*	
H3B	0.3791	1.2281	0.1212	0.058*	
C4	0.1875 (4)	1.1231 (3)	0.16346 (19)	0.0449 (8)	
H4A	0.2283	1.1194	0.2166	0.054*	
H4B	0.1151	1.2000	0.1602	0.054*	
C5	−0.0354 (4)	0.9767 (4)	0.19972 (18)	0.0491 (8)	
H5A	0.0010	0.9678	0.2529	0.074*	
H5B	−0.0935	0.8958	0.1853	0.074*	
H5C	−0.1019	1.0562	0.1955	0.074*	
C6	0.5641 (4)	1.0490 (4)	0.0483 (2)	0.0628 (11)	
H6A	0.6305	0.9691	0.0477	0.094*	
H6B	0.6241	1.1279	0.0643	0.094*	
H6C	0.5224	1.0643	−0.0034	0.094*	
C7	0.1282 (4)	0.7396 (3)	0.1223 (2)	0.0580 (9)	
H7A	0.0840	0.7508	0.0708	0.087*	
H7B	0.0458	0.7209	0.1593	0.087*	
H7C	0.2011	0.6642	0.1219	0.087*	
Cl1	0.44725 (9)	0.46036 (8)	0.01637 (4)	0.0479 (2)	
Cl2	0.42671 (10)	0.48120 (12)	0.22655 (4)	0.0642 (3)	
Cl3	0.70714 (11)	0.70748 (9)	0.11735 (6)	0.0600 (2)	
Cl4	0.79167 (12)	0.34029 (10)	0.13266 (5)	0.0606 (2)	
N1	0.1024 (2)	0.9929 (3)	0.14575 (12)	0.0334 (5)	
N2	0.4314 (3)	1.0266 (3)	0.10512 (14)	0.0390 (6)	
H1A	0.054 (3)	1.003 (3)	0.0967 (16)	0.033 (7)*	
H2C	0.477 (4)	1.023 (3)	0.1532 (18)	0.046 (9)*	

### Atomic displacement parameters (Å^2^)

**Table:**

	*U*^11^	*U*^22^	*U*^33^	*U*^12^	*U*^13^	*U*^23^
Zn1	0.03299 (15)	0.04154 (17)	0.02676 (15)	−0.00188 (15)	−0.00155 (14)	−0.00045 (16)
C1	0.0323 (14)	0.0395 (17)	0.0359 (16)	0.0029 (14)	−0.0043 (14)	0.0082 (12)
C2	0.0345 (16)	0.0368 (17)	0.0390 (15)	0.0024 (13)	0.0001 (13)	−0.0014 (13)
C3	0.055 (2)	0.0371 (18)	0.052 (2)	−0.0065 (15)	0.0007 (17)	0.0019 (14)
C4	0.048 (2)	0.0392 (19)	0.0472 (17)	−0.0059 (15)	−0.0009 (17)	−0.0108 (14)
C5	0.0387 (16)	0.064 (2)	0.0445 (17)	0.0051 (17)	0.0090 (14)	0.0016 (15)
C6	0.045 (2)	0.084 (3)	0.059 (2)	−0.0144 (19)	0.0148 (18)	0.0118 (19)
C7	0.0445 (18)	0.0365 (17)	0.093 (3)	−0.0038 (13)	0.009 (2)	0.008 (2)
Cl1	0.0417 (4)	0.0762 (6)	0.0259 (3)	−0.0185 (4)	−0.0051 (3)	0.0063 (3)
Cl2	0.0424 (4)	0.1219 (9)	0.0283 (4)	−0.0064 (6)	0.0046 (3)	0.0013 (4)
Cl3	0.0541 (4)	0.0453 (5)	0.0806 (6)	−0.0143 (4)	−0.0084 (6)	−0.0038 (5)
Cl4	0.0641 (5)	0.0642 (6)	0.0536 (5)	0.0256 (5)	−0.0113 (5)	−0.0086 (4)
N1	0.0325 (11)	0.0402 (13)	0.0274 (11)	0.0026 (14)	−0.0013 (9)	0.0007 (10)
N2	0.0331 (13)	0.0481 (17)	0.0357 (13)	−0.0095 (11)	−0.0032 (10)	0.0030 (10)

### Geometric parameters (Å, °)

**Table:**

Zn1—Cl3	2.2355 (9)	C4—H4A	0.9700
Zn1—Cl4	2.2597 (9)	C4—H4B	0.9700
Zn1—Cl2	2.2658 (8)	C5—N1	1.499 (3)
Zn1—Cl1	2.2795 (8)	C5—H5A	0.9600
C1—N1	1.513 (4)	C5—H5B	0.9600
C1—C2	1.517 (4)	C5—H5C	0.9600
C1—C7	1.519 (4)	C6—N2	1.504 (4)
C1—H1	0.9800	C6—H6A	0.9600
C2—N2	1.470 (4)	C6—H6B	0.9600
C2—H2A	0.9700	C6—H6C	0.9600
C2—H2B	0.9700	C7—H7A	0.9600
C3—N2	1.485 (4)	C7—H7B	0.9600
C3—C4	1.519 (5)	C7—H7C	0.9600
C3—H3A	0.9700	N1—H1A	0.93 (3)
C3—H3B	0.9700	N2—H2C	0.91 (3)
C4—N1	1.487 (4)		
			
Cl3—Zn1—Cl4	108.34 (5)	N1—C5—H5A	109.5
Cl3—Zn1—Cl2	112.33 (4)	N1—C5—H5B	109.5
Cl4—Zn1—Cl2	112.08 (4)	H5A—C5—H5B	109.5
Cl3—Zn1—Cl1	109.55 (3)	N1—C5—H5C	109.5
Cl4—Zn1—Cl1	110.33 (4)	H5A—C5—H5C	109.5
Cl2—Zn1—Cl1	104.16 (3)	H5B—C5—H5C	109.5
N1—C1—C2	108.4 (2)	N2—C6—H6A	109.5
N1—C1—C7	111.1 (3)	N2—C6—H6B	109.5
C2—C1—C7	109.3 (3)	H6A—C6—H6B	109.5
N1—C1—H1	109.3	N2—C6—H6C	109.5
C2—C1—H1	109.3	H6A—C6—H6C	109.5
C7—C1—H1	109.3	H6B—C6—H6C	109.5
N2—C2—C1	113.2 (2)	C1—C7—H7A	109.5
N2—C2—H2A	108.9	C1—C7—H7B	109.5
C1—C2—H2A	108.9	H7A—C7—H7B	109.5
N2—C2—H2B	108.9	C1—C7—H7C	109.5
C1—C2—H2B	108.9	H7A—C7—H7C	109.5
H2A—C2—H2B	107.7	H7B—C7—H7C	109.5
N2—C3—C4	111.7 (3)	C4—N1—C5	110.3 (2)
N2—C3—H3A	109.3	C4—N1—C1	111.1 (2)
C4—C3—H3A	109.3	C5—N1—C1	113.9 (2)
N2—C3—H3B	109.3	C4—N1—H1A	107.7 (19)
C4—C3—H3B	109.3	C5—N1—H1A	102.4 (16)
H3A—C3—H3B	107.9	C1—N1—H1A	111.0 (18)
N1—C4—C3	111.1 (2)	C2—N2—C3	109.8 (2)
N1—C4—H4A	109.4	C2—N2—C6	111.9 (2)
C3—C4—H4A	109.4	C3—N2—C6	111.6 (3)
N1—C4—H4B	109.4	C2—N2—H2C	111 (2)
C3—C4—H4B	109.4	C3—N2—H2C	107 (2)
H4A—C4—H4B	108.0	C6—N2—H2C	105.5 (19)

### Hydrogen-bond geometry (Å, °)

**Table:**

*D*—H···*A*	*D*—H	H···*A*	*D*···*A*	*D*—H···*A*
N1—H1A···Cl1^i^	0.93 (3)	2.16 (3)	3.092 (2)	177 (3)
N2—H2C···Cl2^ii^	0.91 (3)	2.24 (3)	3.140 (3)	171 (3)

Symmetry codes: (i) *x*−1/2, −*y*+3/2, −*z*; (ii) −*x*+1, *y*+1/2, −*z*+1/2.
